# Study on the Application of Fluorinated Polyimide in the Acidic Corrosion Protection of 3-nitro-1,2,4-trizole-5-one (NTO)-Based Explosive Formulations

**DOI:** 10.3390/polym16121624

**Published:** 2024-06-07

**Authors:** Huanmin Liu, Chongchong She, Jiaming Gao, Kun Chen

**Affiliations:** School of Materials Science and Engineering, Beijing Institute of Technology, Beijing 100081, China; liuhuanmincosmos@163.com (H.L.); 3120195576@bit.edu.cn (C.S.); 18434367844@163.com (J.G.)

**Keywords:** acidic protection, fluorinated polyimide (FPI), 3-nitro-1,2,4-triazol-5-one (NTO), molecular dynamic simulation, interfacial effect, high-energy materials

## Abstract

3-nitro-1,2,4-triazol-5-one (NTO) has been widely used as a kind of insensitive single-compound explosive owing to its excellent balance between safety and explosive energy. To reduce its possible acid corrosion and extend its application to insensitive ammunition, acid protection research on NTO-based explosives is significant. Traditionally, the acid protection effect was evaluated by metal corrosion, which is time-consuming and qualitative. An efficient and quantitative method is desirable for evaluating the acid protection effect and exploring novel protection materials. Herein, a polyimide of 4,4’-(hexafluoroisopropene)diphthalic anhydride (6FDA)/2,2-bis(trifluoromethyl)-4,4-diaminobiphenyl (TFMB) was synthesized by replacing the 4,4’-diaminodiphenyl ether (ODA) monomer with a TFMB monomer to act as an acid-protective coating material for NTO-based explosives. Compared with three other coating materials, polyvinylidene fluoride (PVDF), polyetherimide (PEI), and copolyimide (P84), the fluorinated polyimide exhibits the best acid protection effect. Moreover, a new method was constructed to obtain the pH time-dependent curve in order to evaluate efficiently the acid protection effect of the polymer materials. By the virtue of molecular dynamic simulation (Materials Studio 2023), the interfacial effects of the coating materials with NTO-based explosives were obtained. The study provides an interpretation of the acid protection effect on the molecular level, suggesting that the higher content of fluorine atoms is beneficial for stabilizing the active hydrogen atom of the NTO by forming intermolecular hydrogen bonds.

## 1. Introduction

**Figure 1:**
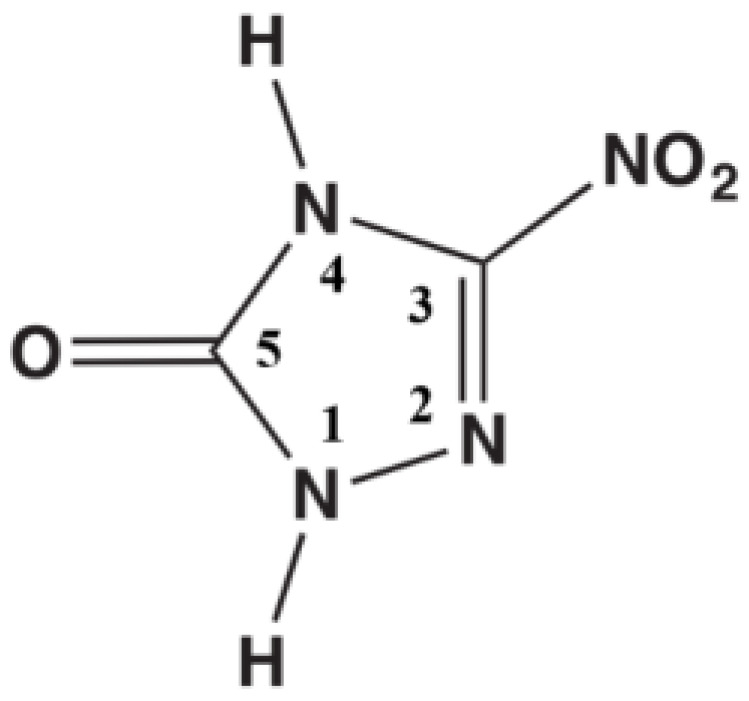
Molecular structure of NTO.

The changing mode of modern warfare and the deteriorating battlefield environment have put forward more stringent requirements on the safety performance of munitions [1]. Improving the insensitivity of ammunition to shock (from bullets, fragments, shaped charge jets, and adjacent detonating munitions), heat (from fires or adjacent thermal events), and/or mechanical effects (impact or drop) has become an inevitable trend in the development of modern ammunition. Modern insensitive munitions should not only meet the technical requirements of detonation ability, but also require insensitive characteristics during the whole life cycle of the munitions [2,3,4]. 3-nitro-1,2,4-triazol-5-one (NTO) exhibits excellent detonation performance as well as superior safety performance as one of the representatives of modern insensitive explosives. The crystal density of NTO is 1.93 g/cm^3^ and its theoretical detonation velocity is 8560 m/s, which is slightly lower than that of cyclotetramethylenetranitramine (HMX), close to that of cyclotrimethylene trinitramine(RDX), and 7% higher than that of 1,3,5-trinitro-2,4,6-triaminobenzene (TATB) [5]. In terms of safety performance, the impact sensitivity and the friction sensitivity are greater than 280 cm (U.S. Type 12 drop hammer) and 353 N (U.S. SNPE), respectively, which are significantly higher than those of HMX and RDX and close to TATB [6]. Due to its balance between power and safety, NTO has been used in the formulations of IMX-101 and IMX-104. However, the pK_a_ value of NTO is 3.76, which is more acidic than acetic acid (pK_a_ = 4.67) and less acidic than chloroacetic acid (pK_a_ = 2.86) [7]; thus, NTO exhibits acidity. The nitro and carbonyl groups in the NTO molecule have strong electron-withdrawing effects, resulting in a decrease in the electron density of the nitrogen atoms at the 1- and 4-position (Figure 1). As a result, the proton on the nitrogen of 4-position is more easily ionized, and may result in the corrosion of the materials in contact with NTO in practical applications. Therefore, exploring acid-protection materials is significant for the application and hazard protection of NTO-based formulation explosive.

Encapsulation in polymer materials is a common strategy for preventing acidic corrosion [8,9,10]. The coating effects of vinylidene fluoride–tetrafluoroethylene–hexafluoropropylene copolymer (F246G), polystyrene (PS) [8], polyurethane (PU) [9], and Fluor elastomer F2311 [10] have been reported. Although efforts have been devoted to exploring protection materials and controlling the encapsulating process, the interpretation on the molecular level and quantitative expression of the protective effect are still obscure, which are significant for improving the evaluation efficiency and designing novel acid protection materials. Polyimide (PI) commonly shows excellent mechanical properties and good stability at both high and low temperatures, and is thus widely used in high-temperature-resistant ultra-thin filter membranes, optoelectronic functional materials, engineering plastics, and the aerospace and military industries [11,12,13,14,15,16]. The presence of imide groups within the PI material makes it inherently resistant to both high and low temperatures and exhibits excellent mechanical properties. Nevertheless, the weak solubility of the materials containing imide groups leads to poor processability; charge transfer complex (CTC) may occur within or between PI molecules during the synthesis leading to a reduction in the thermal and mechanical properties [17,18,19,20,21,22]. Due to the small radius of fluorine atoms, low molar polarization rate, and large free volume of representative fluorine groups -CF_3_, introducing fluorine atoms effectively reduces the stacking efficiency, increases the intermolecular spacing, and reduces the intermolecular interaction forces, thus improving the solubility of the polymer and reducing the dielectric constant, which makes the spray coating process more convenient. The fluorine atom is highly electronegative and the C–F bond is very firm, which can reduce the formation of CTC in PI and enhance the high thermal stability. In addition, the low surface energy of fluorine atoms makes them strongly hydrophobic, which reduces the moisture absorption of the film. These advantages make the fluorinated polyimide (FPI) materials with high fluorine content a suitable candidate for NTO acid protective coating materials [21,23].

**Figure 2:**
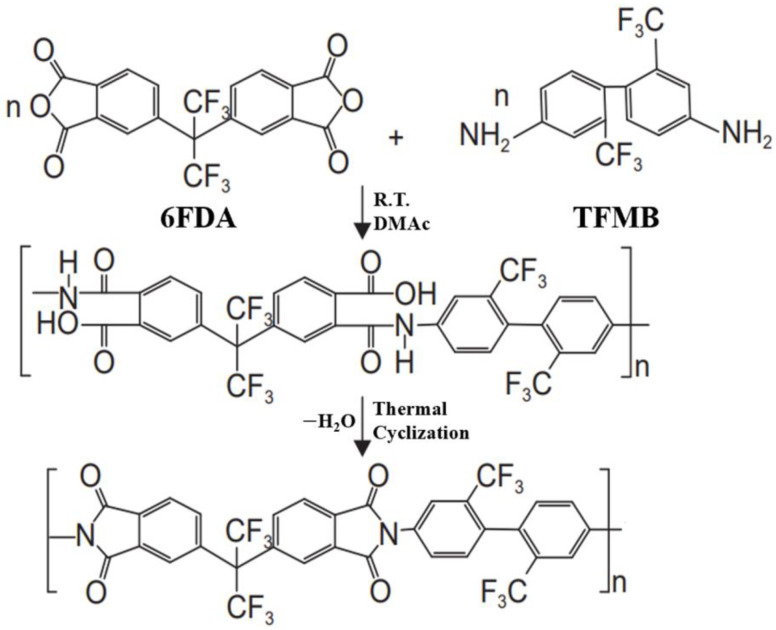
Schematic diagram of the synthesis process of fluorinated polyimide.

Herein, the TFMB monomer was introduced to partly replace the ODA monomer in the traditional fluorinated polyimide 6FDA/ODA to increase the fluorine content (Figure 2). A series of FPIs with different fluorine contents were synthesized and characterized to demonstrate the improvement of hydrophobicity, mechanical properties, and dielectric properties of FPI materials by increasing the fluorine content. The synthesized FPI materials were sprayed on the pressed NTO-based explosive formulation PNH (50 wt.% NTO, 44 wt.% HMX, 7 wt.% binder). The encapsulated column was immersed into water and the time-dependent pH values were recorded to evaluate the acid protection effect of the polymers. Besides FPI, the protection effects of polyvinylidene fluoride (PVDF), polyetherimide (PEI), and benzotetracarboxylic acid dianhydride (BDTA)– diisocyanate diphenylmethane ester (MDI)–toluene diisocyanate ester (TDI) copolyimide (P84) were also recorded. Our observations indicated that the FPI with high fluorine content provides a superior proton barrier effect. In order to interpret the acid-protection process of NTO-based explosives and describe the interfacial effect quantitatively, the binding energy, cohesion energy density, and mechanical properties between coating materials and NTO-based explosives were simulated by Materials Studio 2023 (MS). The simulation results provide the interpretation of the acid protection coating effect of FPI on NTO-based explosives.

## 2. Materials and Methods

### 2.1. Materials

Polyvinylidene fluoride (PVDF, AR, CAS24937-79-9) was purchased from Shanghai Solvay Chemical Technology Co. Ltd. (Shanghai, China). Polyetherimide (PEI, AR, CAS61128-4-9) was purchased from Shanghai Tengyi Chemical Technology Co., Ltd. (Shanghai, China). Copolyimide (P84, AR, CB23309706) was purchased from Bohai Chemical Technology Co., Ltd. (Shanghai, China). 2,2-bis(trifluoromethyl)-4,4-diaminobiphenyl (TFMB, AR, CAS341-58-2),4,4′-(hexafluoroisopropene) diphthalic anhydride (6FDA, AR, CAS1107-00-2), and 4,4’-diaminodiphenyl ether (ODA, AR, CAS101-80-4) were purchased from Yangguang Chemical Technology Co., Ltd. (Changzhou, China). N, N-dimethylformamide (DMF, CAS624-49-7) N, N-dimethylacetamide (DMAc, CAS127-19-5), and -N-methylpyrrolidone (NMP, CAS870-52-4) were purchased from Beijing Tongguang Fine Chemical Co., Ltd. (Beijing, China).

### 2.2. Synthesis of Fluorinated Polyimide

The designed synthesis process of fluorinated polyimide molecules with high fluorine content is shown in Figure 2. By introducing a TFMB monomer into the polyimide PI of 6FDA/ODA and adjusting the fluorine content by controlling the ratio of ODA to TFMB, a series of fluorinated polyimide FPI film materials were prepared and the effect of this design idea on the actual material properties was characterized by various characterizations.

The preparation steps of fluorinated polyimide films mainly include synthesis of fluorinated polyamide acids, film coating, and thermal cyclization. In order to verify the effects of the TFMB monomer on various properties of the polyimide system of 6FDA/ODA system, homopolymerization or copolymerization systems with different molar ratios (10:m:n) are listed in Table 1, and the specific processes were as follows.

For instance, in the synthesis of FPI-3 (ODA:TFMB = 5:5), ODA (2.51 g, 23.0 mmol) and TFMB (7.41 g, 23.0 mmol) were added into a dry 250 mL three-necked flask. Then, 100 mL of N, N-dimethylacetamide (DMAc) was added into the flask, and the solution was stirred for about 20 min under ice bath conditions and nitrogen protection. The monomer 6FDA (20.44 g, 46.0 mmol) was added to the above solution in batches while stirring (the molar ratio of dianhydride to diamine was 1:1), and the synthesis volume of polyamide acid solution used in each experiment was 100 mL, and the solid content of the solution was 20%. Other polymers were obtained by adjusting the amount of ODA and TFMB.

**Figure 3:**
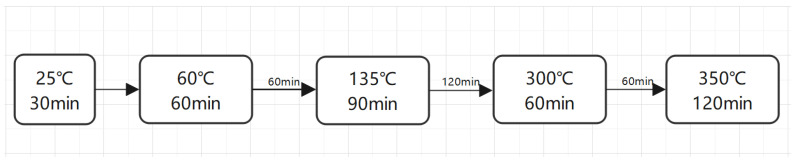
The thermal cyclization process.

The prepared polyimide acid was deposited on a flat glass substrate, and the glass substrate was placed in the ultra-clean bench. After the surface of the film is completely dry, it was placed in an electric blast drying oven for thermal cyclization. The thermal cyclization process is shown in Figure 3.

### 2.3. Characterizations

#### 2.3.1. Infrared Spectroscopy Measurement

The prepared samples were tested by infrared spectroscopy using NICOLET’s NEXUS-670 with a scan wave number of 4000–400 cm^−1^.

#### 2.3.2. Dielectric Constant Measurement

The prepared fluorinated polyimide films were cut into square pieces with a size of 15 mm × 15 mm, and the thickness was measured using a digital thickness gauge. Later, gold was sprayed on both surfaces using an ion sputterer to form 10 mm × 10 mm square gold electrodes. The dielectric properties were measured at room temperature using a precision impedance analyzer (AGILENT 4294A) in the frequency range of 40 Hz–110 MHz. The dielectric loss tanδ of the films was derived directly from the measurements, and the dielectric constant ε was calculated from Equation (1):

(1) $$\varepsilon =\frac{C\times d}{\varepsilon_{0}\times S}$$

where: ε represents the dielectric constant, *C* represents the measured capacitance, *ε*_0_ represents the vacuum dielectric constant (8.854 × 10^−12^ F/m), *d* represents the film thickness (the thickness needs to be measured before gold spraying), and *S* represents the area of the gold-sprayed square.

#### 2.3.3. Mechanical Properties Measurement

The polymer film was cut into standard dumbbell-shaped samples with a standard cutter, the film thickness was measured and recorded using a digital thickness gauge, and then the mechanical parameters (tensile strength, modulus, elongation at break) of the samples were measured on a SANSCMT4104 universal mechanical testing machine at a tensile rate of 10 mm/min at room temperature [24].

#### 2.3.4. Hydrophobic Property Measurement

The contact angle of the film was measured using a Dataphysics OCA15D contact angle tester with water as the test solution at room temperature.

### 2.4. Experimental Study of Proton Release Pattern in Water for PNH Formulation Columns with FPI and Different Coating Materials Sprayed

**Figure 4:**
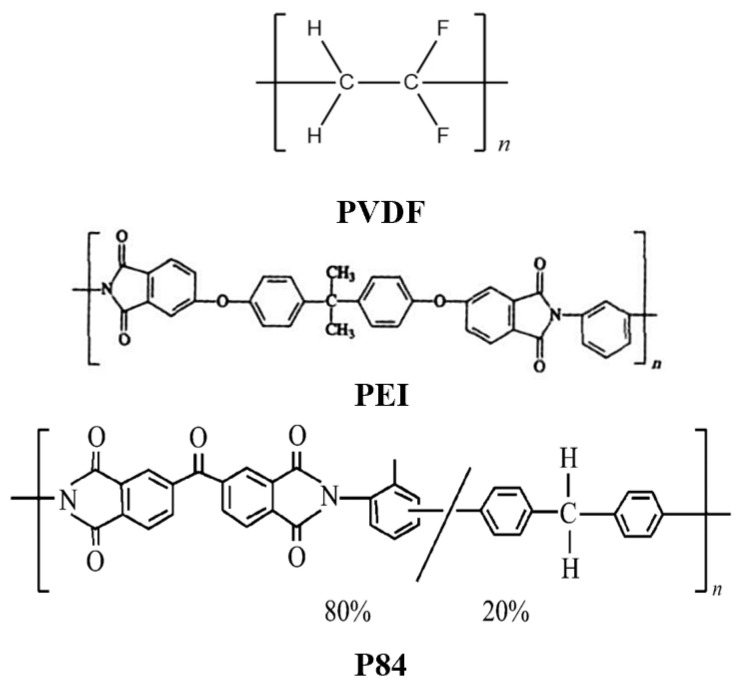
Molecular structures of PVDF, PEI, and P84.

In order to quantitatively evaluate effects of polymers on the acidic protection of NTO-based explosive formulations, the synthesized FPI with different fluorine content and common soluble high-temperature-resistant coating materials PVDF, PEI, and P84 were sprayed onto PNH columns. Then, the encapsulated columns were immersed into pure deionized water, and the time-dependent pH values were recorded. The molecular structures of PVDF, PEI, and P84 are shown in Figure 4.

#### 2.4.1. Vacuum Stability Compatibility Test

In order to ensure the safety of the intact experimental process, the compatibility between the component of PNH formulation and the encapsulating materials was tested prior to the actual experiments. According to the compatibility test method, the paper adopts the vacuum stability test pressure sensor method to test the compatibility of NTO and binder.

Reagent dosage: a single sample of explosive or encapsulating polymer is 2.50 ± 0.01 g; a mixed sample is 5.00 ± 0.01 g; mixed mass ratio 1:1; test temperature was 100 ± 0.5 °C; heating time was 48 h. Compatibility is calculated according to Formula (2):

(2) $$R=V_{C}-(V_{A}+V_{B})$$

In the formula: *R*—reaction net increase outgassing volume, mL;

*V_C_*—The amount of outgassing of the mixed sample, mL;

*V_A_*—Degassing volume of explosive sample, mL;

*V_B_*—The amount of outgassing of the contact material, mL.

Recommended grade for evaluating compatibility:

|*R*| < 3.0 mL is compatible;

|*R*| = 3.0~5.0 mL shows a moderate response;

|*R*| > 5.0 mL is not compatible.

#### 2.4.2. Measurement of Time-Dependent pH Curves of PNH Columns with Different Coatings

**Figure 5:**
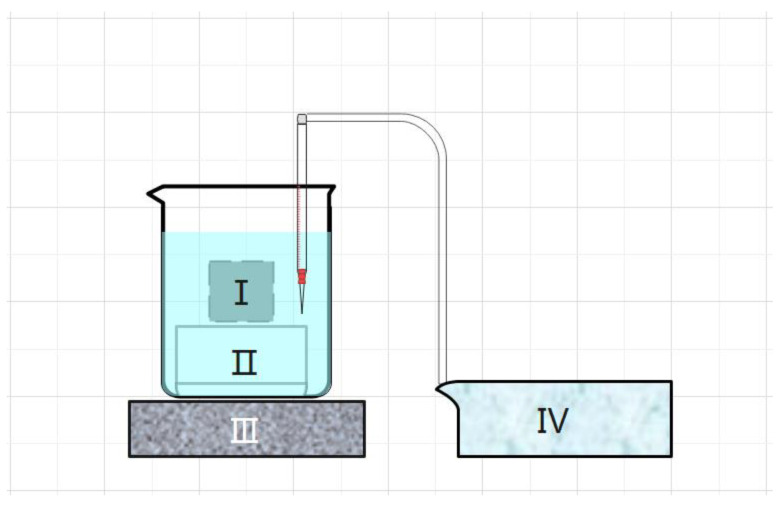
Schematic diagram of the experimental setup for testing the proton release law of PNH explosive columns. Ⅰ. 20 mm × 20 mm PNH column (sprayed with different polymer coatings). Ⅱ. PMMA support frame. Ⅲ. Magnetic stirrer. Ⅳ. pH meter.

The schematic diagram of the experimental setup for testing the proton release law of PNH explosive columns is shown in Figure 5. The experimental steps were as follows:

(1) PNH formulation modeling powder was pressed into a 20 mm high × 20 mm diameter explosive column, then the column was put into 500 mL of pure deionized water and the pH values of the solution at different times were measured with a pH meter;
(2) The encapsulating polymer was dried in a vacuum oven for at least 72 h and then prepared with the corresponding solvent as 10% (wt.%) solution. DMF was chosen for dissolving FPI, PVDF, and P84. NMP was chosen for dissolving PEI. The solution was stirred for 10 h at a heating temperature of 60 °C to obtain a completely dissolved, clarified, and transparent solution;
(3) The configured polymer solution was sprayed evenly on the surface of the explosive column by vacuum spraying equipment, and the cladding thickness was controlled at ca. 0.1 mm after drying at room temperature for 6 h;
(4) The encapsulated columns were put into 500 mL of deionized water, and the time-dependent pH values of the solution were recorded.

#### 2.4.3. Study of Metal Corrosion of NTO-Based Insensitive Explosive Formulation Columns Coated with Polymer Film Materials

In order to verify the actual corrosion effect of the munition casing after the polymer protective layer is wrapped around the explosive column, the munition casing steel material was made into a Φ25 mm × 2 mm circular sheet, and the oxidized layer was ground off with an emery cloth to ensure that the roughness R_a_ ≤ 3.2 μm, and then it was fixed in the middle of the two spray-coated explosive columns. The steel sheet sandwiched by two columns was placed at 60 °C and a humidity of 80%rh for 30 days to evaluate the acid protection effect of the polymers.

Six groups were set up for the munition casing metal corrosion experiment. The contents are shown in Table 2 below. 

### 2.5. Molecular Dynamics Simulation of the Interfacial Effect with Materials Studio 2023

In order to clarify the interfacial effect of polymer coatings on the acid protection of NTO from a microscopic molecular perspective, the binding energy, cohesive energy density, and mechanical properties of the coating materials and PNH were simulated by Materials Studio 2023 (MS). The coating connection mechanism between the polymer and the explosive was explored.

The force field of COMPASS, the convergence energy levels of fine structures, the electrostatic force of Ewald and the atom-based van der Waals force are adopted, respectively. The detailed simulation procedure can be found in the Supplementary Information (SI).

## 3. Results and Discussions

### 3.1. Synthesis and Characterization of Fluorinated Polyimide Materials

The synthesized polymer materials were characterized to demonstrate the effect of the fluorine content on their properties after preparing a series of fluorinated polyimide materials.

#### 3.1.1. FTIR Spectrum

**Figure 6:**
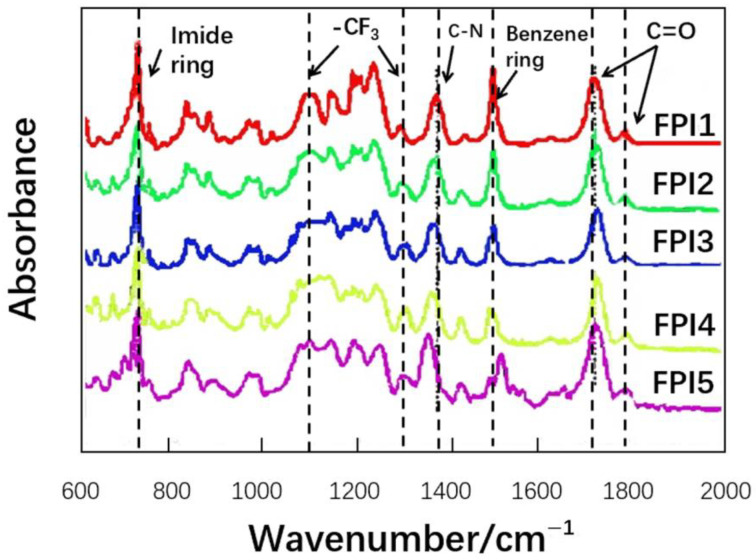
Comparison of FTIR spectra of fluorinated polyimide materials with different TFMB molar ratios.

The IR spectra of FPI1 to FPI5 are shown in Figure 6 and the assignments of the characteristic peaks are listed in Table 3. According to the conventional IR detection method for polyimide polymers, the characteristic peaks at 1775 cm^−1^ (asymmetric stretching vibration of the carbonyl group of the imide ring C=O), 1715 cm^−1^ (symmetric stretching vibration of the imide ring C=O), 1350 cm^−1^ (stretching vibration of the C–N bond vibration), and 735 cm^−1^ (in-plane bending vibration of the imide ring C=O) were observed, which strongly indicated the structure of polyimide (PI). No absorption peaks caused by N-H stretching in the raw material were observed around 3345~3356 cm^−1^, which indicates that all the amino groups experienced acylation and were converted to imide groups [25].

In the series of FPI1~FPI5, with the increase in TFMB monomer content and the increase in trifluoromethyl structure in the polymer structure, multiple peaks can be observed in the range of 1055~1305 cm^−1^, which are attributed to the vibration of -CF_3_, indicating the successful preparation of the fluorinated polyimide materials.

#### 3.1.2. Dielectric Constant

**Figure 7:**
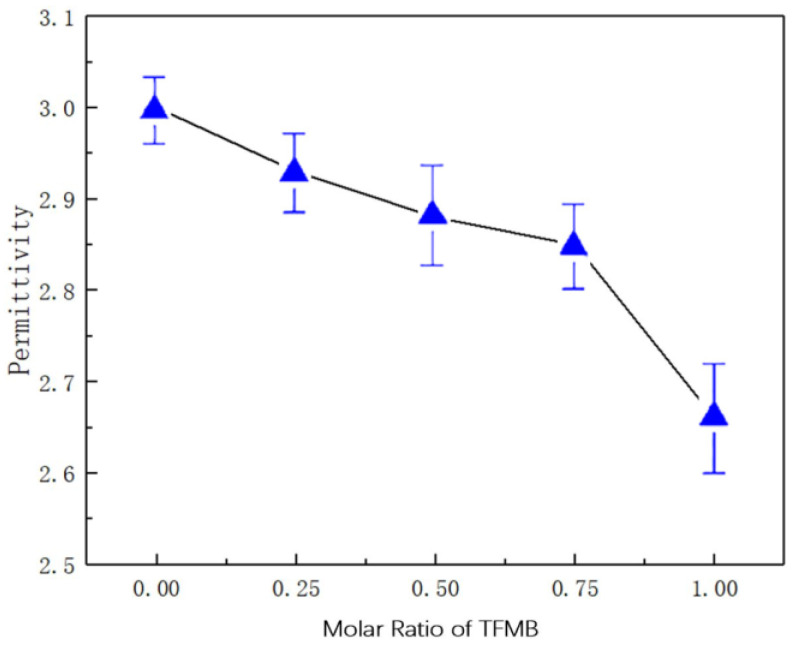
Comparison of dielectric constant ε of fluorinated polyimide materials with different TFMB molar ratios.

The dielectric constants of FPI1~FPI5 were measured at the frequency of 1 MHz and are shown in Figure 7. As the proportion of TFMB monomers increased, a decrease in the dielectric constant of the material was observed. Since the dianhydride monomer 6FDA contains two trifluoromethyl structures, every imine unit possesses four trifluoromethyl groups. FPI5 contains the highest amount of fluorine, and showed a dielectric constant of around 2.69 C^2^/(N∙M)^2^. This observation can be attributed to the increased fluorine content of the polymers. Due to the strong electronegativity of fluorine atoms, the dipole polarization ability of C–F bonds is smaller than the imide group. Thus, the overall dipole moment of the polyimides was reduced after introducing the fluorine group and the dielectric constant was also reduced. At the same time, it is more difficult for protons to pass through the FPI material owing to the strong electronegativity of fluorine atoms, which is beneficial for the acid corrosion protection performance of FPI material. It suggested that the dielectric constant, as a crucial research direction for the modification of polyimide materials, negatively correlated with the acid protection performance.

#### 3.1.3. Mechanical Property Tests

The mechanical properties of polymer coating materials are also important parameters. The mechanical properties of FPI materials are shown in Table 4. Among this series of FPI polymers, homopolymerized FPI showed higher mechanical strength and elongation at break. The tensile strength (122.14 ± 1.77 MPa), tensile modulus (2.06 ± 0.09 GPa), and elongation at break (8.44 ± 0.11%) of FPI5 exceeded those of FPI1 (107.44 ± 2.55 MPa, 2.02 ± 0.14 GPa, and 8.16 ± 0.08%). At the same time, the tensile strength and elongation at break of copolymerized FPIs were lower than those of homopolymerized FPIs, which may be attributed to the fluorine-containing structure in 6FDA units and the synergistic effect of TFMB units. Comparing a copolymerized FPI, a homopolymerized FPI promotes the ordered stacking of molecular chains to a certain extent, and thus improves the mechanical performance of the polymer. In general, the modulus represents the ability of a material to resist deformation, and the chemical bond nature, chemical bond density, and intermolecular forces mainly determine the magnitude of this ability, which in turn is directly influenced by the free volume size. Therefore, the reduction in free volume size is one of the important factors for a better mechanical modulus of the material. It can be seen that the designed fluorinated polyimides exhibit high modulus, and the mechanical properties first decreased and then increased with the addition of the TFMB monomer. After the ODA monomer is completely replaced by the TFMB monomer, the mechanical properties will return to an even higher level.

#### 3.1.4. Hydrophobicity Test

It is well known that the pK_a_ value of organic compounds is related to their solution environment. The protection film may absorb moisture from the surrounding environment during the use process, which may make the mechanical and electrical properties of the material decrease during the use process, and results in a reduction in acid corrosion protection. Polymer materials with strong hydrophobicity will reduce the amount of water absorption from the environment, and the diffusion coefficient of protons in the polymer material will increase with the increase in water content, resulting in the decline of acid resistance. The moisture absorption of the film is related to the hydrophobicity of the material itself and the intermolecular chain force.

**Figure 8:**
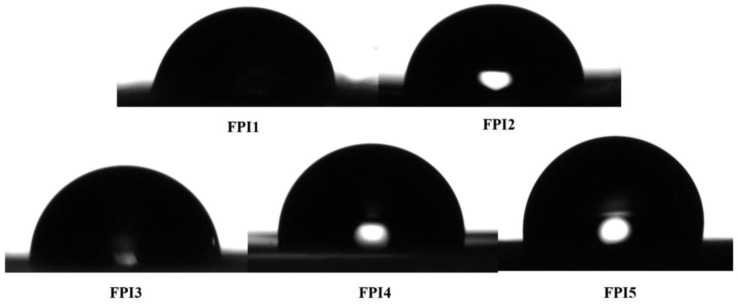
Comparison of contact angle of fluorinated polyimide materials with different TFMB molar ratios.

To evaluate the hydrophobicity of the FPI films, their contact angles were measured. As the TFMB content increases, the fluorine content of the material increases, providing more acceptors of hydrogen bonds. As shown in Figure 8 and Table 5, the contact angle of water on the surface of FPI film increases from 77.1 ± 0.88° in FPI1 to 103.60.76° in FPI5, which indicates the elevated hydrophobicity of the film. Since the hydrophobicity is determined by the chemical structure of the material and the intermolecular chain force is determined by the hydrogen bonding content, the degree of cross-linking, as well as the molecular polarity, it is reasonable that the hydrophobicity of the films was elevated as the TFMB unit gradually replaced the ODA unit along with the elevation of the hydrogen bond acceptor.

Among all the fluorinated polyimide materials with different TFMB molar ratios, FPI5 exhibited the lowest dielectric constant, the best mechanical properties, and the largest contact angle, which are all favorable for acid protection. Thus, FPI5 material was selected as the protection material in the subsequent study.

### 3.2. Experiment on the Release Pattern of Protons from PNH Explosive Formulations Coated with FPI and Different Materials

#### 3.2.1. Compatibility

The results of the vacuum stability test were used to evaluate the compatibility of the systems, which ensured safety during the experiments and applications. As shown in Table 6, the absolute value of R for each PNH/coating material and coating material/munition shell system is less than 3.0 mL according to the vacuum stability test method, which indicates that the compatibility of these systems is acceptable.

#### 3.2.2. Experimental Study of the Proton Release Pattern of PNH/Coating Material Systems

**Figure 9:**
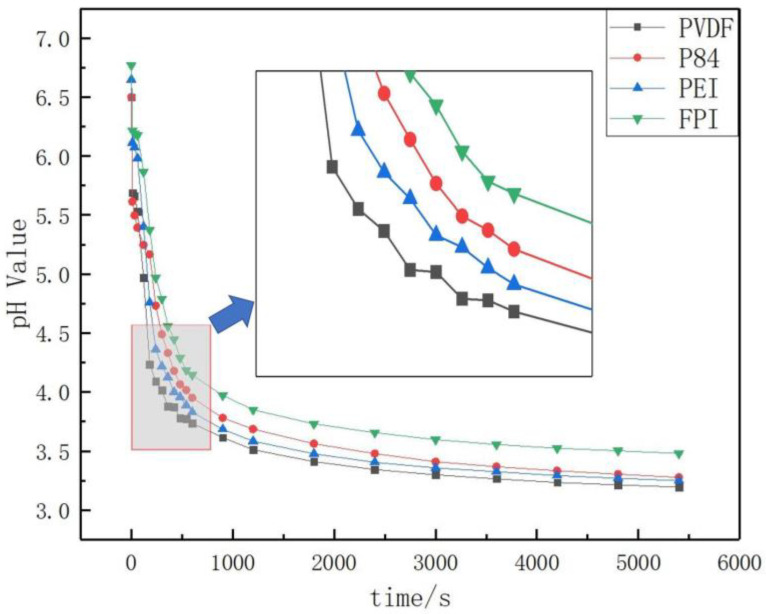

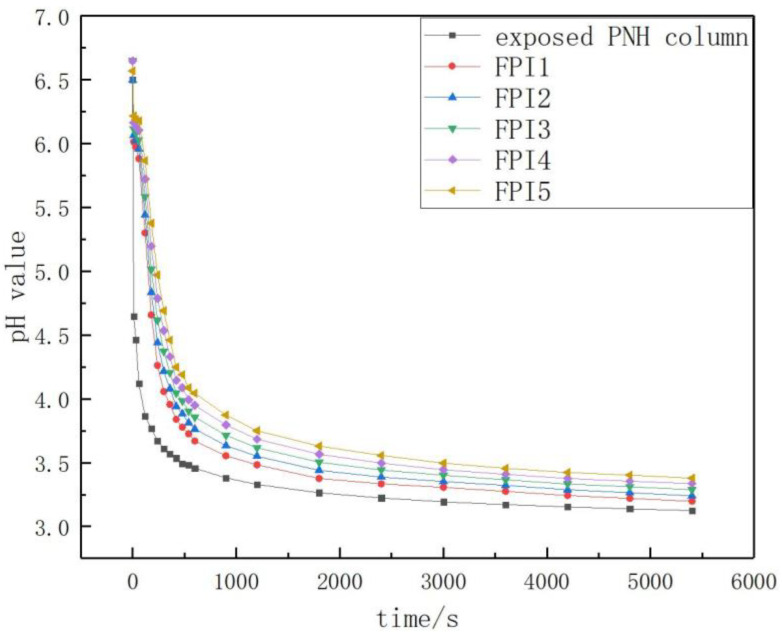
The experimental proton release pattern of PNH/coating material systems.

The proton release curves were measured by placing the PNH columns encapsulated in FPI, PVDF, P84, and PEI in 500 mL of pure deionized water, respectively. The time-dependent pH values are shown in Figure 9.

The pH value of deionized water in each experimental device system was 6.5 at the beginning of the experiment, and as the experiment proceeded the protons of the NTO molecule in the explosive gradually dissociated and diffused into the deionized water, leading to a decrease in pH value. The pH of the deionized water in the experimental system with PVDF coating was reduced to 3.7 at 500 s and to 3.2 after 5000 s. The pH of the deionized water in the experimental system coated with PEI and P84 was about 3.9 at 500 s and about 3.3 after 5000 s. The pH values of the deionized water in the experimental system coated with FPI were about 4.1 at 500 s and about 3.6 after 5000 s.

The experimental proton release curve measured from the PNH column encapsulated in FPI showed its superior proton barrier efficiency compared with the comparative material, which demonstrates that the prepared FPI material with a high fluorine content does show a better protective effect. When the PNH columns were encapsulated in PVDF, having a lower fluorine content and a simple structure, the experiment showed that the pH value decreased fastest, indicating the acid protection effect of PVDF is weak. This observation may be attributed to the lack of amide bonding in the molecular structure of PVDF and its relatively inferior mechanical properties, which led to a loose packing structure and a worse sealing effect during the encapsulation process. The relatively higher content of amide bonds in the chemical structure of P84 resulted in better protection.

To furtherly confirm the effect of fluorine on acid protection, the explosive columns were encapsulated in FPI materials with different fractions of TFMB. As the ratio of TFMB to ODA gradually increased, the proton release curves of FPI1 to FPI5 show that the protective effect was enhanced, which verifies the positive relationship between the content of fluorine and the final protective ability of FPI materials, and may be attributed to the possibility that bonds between hydrogen and fluorine retard the diffusion of protons across the polymer film.

#### 3.2.3. Experimental Study of the Metal Corrosion Effect of PNH/Coating Material Systems

**Figure 10:**
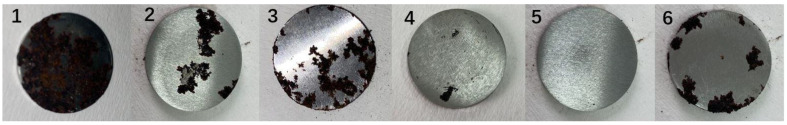
Metal corrosion effect experiments on ammunition shell material samples by PNH explosive column coated with no coating (**1**), PVDF (**2**), PEI (**3**), P84 (**4**), PFI5 (**5**), and shellac varnish (**6**).

The results of the metal corrosion experiments are shown in Figure 10. Based on the level of corrosion of the metal sheet, it is evident that the PNH formulation does have a significant corrosive effect on the ammunition shell material. While PVDF, PEI, P84, FPI, and shellac show a certain degree of protection, FPI exhibits the best protective effect, with no corrosion occurring at all. The observation is consistent with the proton ion release measurements, indicating the experiment can be used to determine the acid protection effect of the polymer.

### 3.3. Molecular Simulation Study of Interfacial Effects of PNH/Polymer Coating Cladding Systems

**Figure 11:**
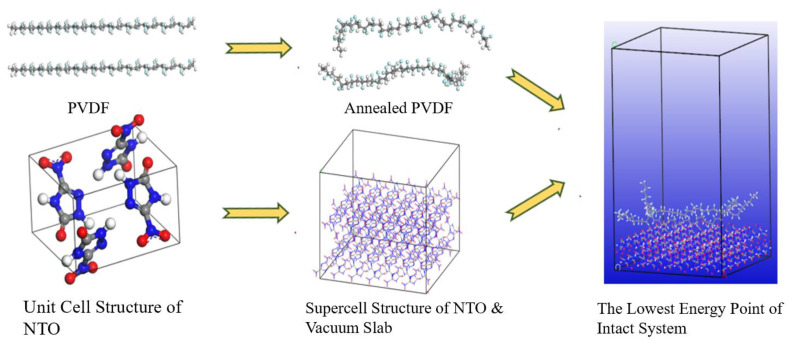
The molecular dynamics simulation process for optimal coating material.

In order to quantify the protective and bonding effects of the coating materials from a microscopic molecular perspective, the binding energy, cohesion energy density, and mechanical properties of PVDF, PEI, P84, and FPI with NTO and HMX in PNH were simulated by molecular dynamics using Materials Studio 2023 (MS), The simulation process is shown in Figure 11.

The binding energy suggested the coating’s ability to encapsulate and bond to the main explosive [26]. The cohesion energy density is commonly used to evaluate the magnitude of intermolecular forces and mainly reflects the interactions between groups [27,28]. Characterizing the energy required to change the state of polymers, the cohesion energy density can also be used to determine the basis of stability of the system. The mechanical properties of the interfacial structure of the explosive and polymer are also directly related to the firmness of the encapsulation. The relative ease of volume change and shape change of the NTO/binder interfacial structure calculated as the ratio of bulk modulus to shear modulus, K/G, can be used to evaluate the encapsulation process [29,30,31,32,33,34]. The mechanical properties of the interface between the coating material and the explosive column improve with the higher K/G value of the interface [35].

**Figure 12:**
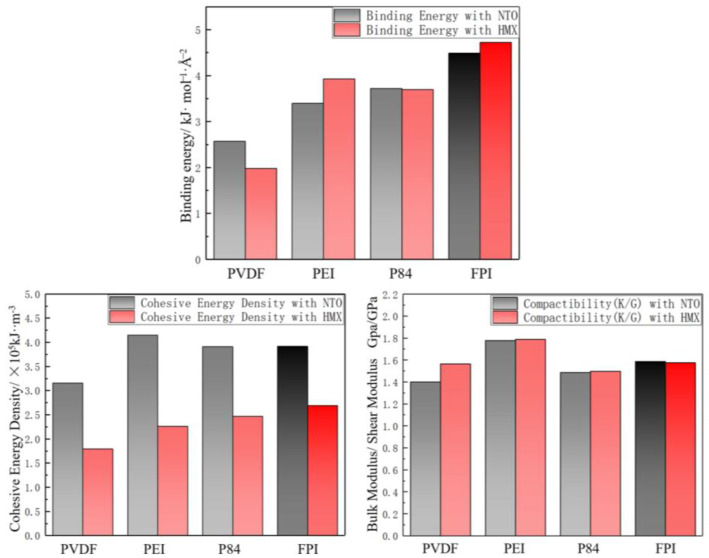
The molecular dynamics simulation results for optimal coating material.

As shown in Figure 12, the binding energy of two materials, P84 and PEI, with the main explosive in the PNH formulation was significantly better than PVDF, and the FPI material showed the best binding energy with NTO and HMX of the four materials. The cohesive energy density of all four materials with NTO is obviously greater than that of HMX. FPI, PEI, and P84 materials are close to the level of cohesive energy density with the main explosive, but all are also superior to PVDF. The mechanical properties of the interfacial structure between PEI coating material and explosive are the most excellent, FPI material is the second, and PVDF and P84 are both inferior. In summary, the bond energy, cohesion energy density, and mechanical properties of PEI and P84 with the main explosive are better than PVDF, with bond lengths between 1.5 Å and 3 Å, which is within the radial distribution function (RDF) of intermolecular hydrogen bonds. Polyimide materials do have outstanding overmolding bonding.

The binding energy, cohesive energy density, and mechanical properties of the FPI to the main explosive are excellent and significantly better than PVDF without amide bonds. The properties are similar to those of P84 with the same amide bond content. This proves that the bonding effect between the fluorinated polyimide material and the explosive column is guaranteed.

**Figure 13:**
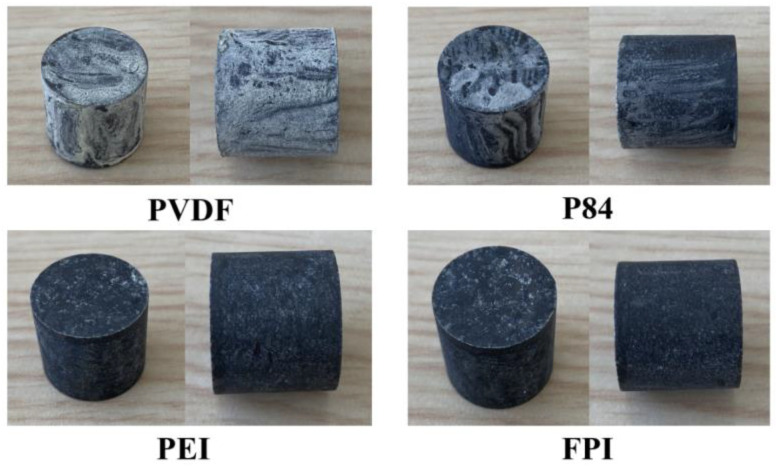
Appearance of the explosive column after being coated with PVDF, P84, PEI, and FPI.

As shown in Figure 13, the morphology of the different protective films on the surface of the explosive column corresponds to the molecular dynamics simulation results as well. PVDF has the most inhomogeneous morphology after spraying due to its poor binding energy, cohesive energy density, and mechanical properties at the interface with the main explosive in the PNH formulation.

Among the three polyimide materials, P84 is a copolymer made of benzophenone tetracarboxylic acid dianhydride (BDTA), diisocyanate diphenylmethane ester (MDI), and diisocyanate toluene ester (TDI); more monomer types lead to easy stress concentration and thus the appearance of silver lines in the protective film. Both FPI and PEI obtained satisfactory performance in molecular dynamics simulations, which led to the formation of a uniformly dense and flat protective layer on the surface of the explosive column.

## 4. Conclusions

In this work, a series of fluorinated polyimides with different fluorine contents were prepared by replacing the ODA with a TFMB monomer in the 6FDA/ODA polyimide system. With the gradual increase in TFMB monomer content, the dielectric properties, mechanical properties, and hydrophobicity of fluorinated polyimide materials have been improved.

The NTO-based explosive column was coated with different polymer materials, and the proton release law was tested. The experimental results prove that FPI5 (6FDA/TFMB) showed the best protection performance among the materials with different ratios of 6FDA/TFMB/ODA, PVDF, PEI, and P84, which is also confirmed by the metal corrosion experiment.

The interfacial effect of binding energy, cohesive energy density, and mechanical properties between the various materials and the explosive were simulated using Materials Studio 2023, and the simulation results confirmed the correlation between the fluorine content and the coating effect, indicating that a high fluorine content is favorable for acid protection owing to the hydrophobicity and the possible interaction between the fluorine and protons. The results are not only significant for the application of NTO-based explosives but also shows a practical implication of evaluating the acid protection of the encapsulating energetic materials.

## Figures and Tables

**Table 1:** Compositions of fluorinated polyimide samples.

Sample	Molar Ratio		
	6FDA	ODA	TFMB
FPI1	10	10	0
FPI2	10	7.5	2.5
FPI3	10	5	5
FPI4	10	2.5	7.5
FPI5	10	0	10

**Table 2:** The contents of metal corrosion experiments of NTO-based explosive formulation columns coated with different polymer film materials.

Experimental Number	Experimental Content
1	Exposed explosive column
2	Explosive column coated with PVDF
3	Explosive column coated with PEI
4	Explosive column coated with P84
5	Explosive column coated with FPI
6	Explosive column coated with shellac varnish

**Table 3:** Wave number distribution of characteristic absorption peaks in FTIR spectra of fluorinated polyimide materials.

Functional Group	Wavenumber/cm^−1^
C=O asymmetrical stretching of imide groups	1775
C=O symmetrical stretching of imide groups	1715
C-N stretching	1350
C=O bending of imide rings	735
-CF_3_	1055~1305
Benzene rings	move from 1509 to 1490 cm^−1^ and strengthen proportionally due to the electron withdrawing groups (-CF_3_)

**Table 4:** Comparison of mechanical properties of fluorinated polyimide materials with different TFMB molar ratios.

Sample	Tensile Strength/MPa	Tensile Modulus/GPa	Elongation at Break/%
FPI1	107.44 ± 2.55	2.02 ± 0.14	8.16 ± 0.08
FPI2	97.63 ± 1.98	2.17 ± 0.22	5.21 ± 0.21
FPI3	89.87 ± 1.54	2.27 ± 0.28	4.45 ± 0.16
FPI4	97.14 ± 1.16	2.17 ± 0.16	4.38 ± 0.24
FPI5	122.14 ± 1.77	2.06 ± 0.09	8.44 ± 0.11

**Table 5:** Comparison of hydrophobicity of fluorinated polyimide materials with different TFMB molar ratios.

Sample	Contact Angle with Water/°
FPI1	77.1 ± 0.88
FPI2	81.6 ± 1.12
FPI3	85.2 ± 0.94
FPI4	90.2 ± 2.07
FPI5	103.6 ± 0.76

**Table 6:** Compatibility of coating materials with PHN explosive and commonly used ammunition casing materials.

System	R (mL)	System	R(mL)
PNH + PEI	0.15	Steel + PEI	0.66
PNH + P84	0.49	Steel + P84	0.38
PNH + PVDF	0.81	Steel + PVDF	0.24
PNH + FPI	0.65	Steel + FPI	0.23
Aluminum–Magnesium Alloy + PVDF	0.43	Titanium alloy + PVDF	0.34
Aluminum–Magnesium Alloy + P84	0.49	Titanium alloy + P84	0.53
Aluminum–Magnesium Alloy + FPI	0.35	Titanium alloy + FPI	0.36

## Data Availability

Data are contained within the article and Supplementary Materials.

## Supplementary Materials

The following supporting information can be downloaded at: https://www.mdpi.com/article/10.3390/polym16121624/s1, Figure S1: The cell structures of NTO and HMX; Table S1: Comparison of the lattice parameters of NTO and HMX before and after optimization in the COMPASS force field with the experimental CCDC values. References [36,37,38,39] are cited in the supplementary materials.
