# Supplementary material for: Study on the Application of Fluorinated Polyimide in the Acidic Corrosion Protection of 3-nitro-1,2,4-trizole-5-one (NTO)-Based Explosive Formulations
Source: Polymers (Basel). 2024 Jun 7;16(12):1624. doi: 10.3390/polym16121624 (PMC11207994; doi:10.3390/polym16121624)
Supplement: Supplementary file 1 [file polymers-16-01624-s001.zip › polymers-2993860-supplementary.pdf]

polymers

MDPI

1. The Process of Molecular Dynamics Simulation

The NTO and HMX cell structures were obtained from the Cambridge Crystallographic Data Centre (CCDC), and the cell structures are shown in Figure S1. The initial NTO and HMX crystals were optimized using the COMPASS force field [1], and the cell parameters and their errors before and after optimization are shown in Table S1.

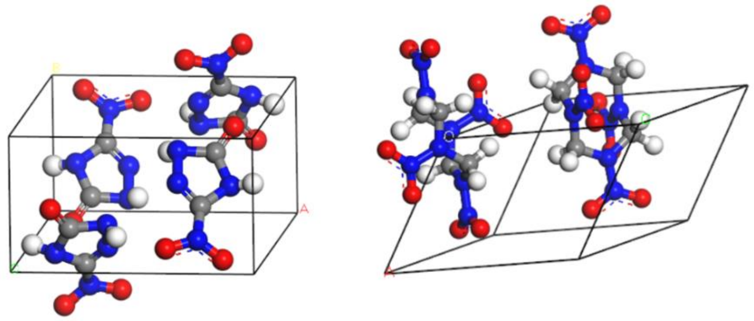

Figure S1. The cell structures of NTO and HMX.

Table S1. Comparison of the lattice parameters of NTO and HMX before and after optimization in the COMPASS force field with the experimental CCDC values.

Lattice Parameters	a/Å NTO	b/Å NTO	c/Å NTO	β/° NTO	a/Å HMX	b/Å HMX	c/Å HMX
Before optimization	9.325	5.450	9.040	101.474	26.160	22.100	26.100
After optimization	9.024	5.147	9.640	94.451	25.176	21.082	27.340
Error%	3.228	5.560	6.637	6.921	3.750	0.460	4.450

From Table S1, the maximum error between the optimized results of NTO and HMX and the experimental results is 0. 07 (β-value of NTO), which is within a reasonable error range, and the COMPASS force field was chosen for the subsequent studies. The molecular models of PVDF, PEI, P84 and the designed FPI were constructed using Materials Studio software, and the polymer chains were geometrically optimized to obtain the stable conformation with the lowest energy.

Modelling the crystalline composite system of polymeric coating materials with NTO and HMX (NTO/PVDF, NTO/PEI, NTO/P84, NTO/FPI, HMX/PVDF, HMX/PEI, HMX/P84, HMX/FPI) . In this paper, NTO/PVDF is used as an example for illustration, as shown in Fig.3 of main part . Firstly, a molecular model of PVDF polymer is constructed . Then the NTO important crystalline surface NTO is extended to supercell . Finally, the polymer PVDF molecular model and NTO supercell are constructed as a composite model. The cell is compressed in 6 directions ± x, ± y, ± z to achieve the theoretical density of the composite system . This method is used in subsequent modelling approaches.

The initial structure of the model is first geometrically optimized before the MD simulation. First, a constant temperature and pressure (NPT) ensemble with Velocity-scaling and Berendsen pressure control [2] is used, and a pressure of 1GPa is selected for the 100ps MD operation to ensure the convergence and stability of the simulated density profile; Then, using the above-mentioned configuration, adopt the same NPT ensemble and temperature and pressure control method, change the pressure to 0. 0001 GPa, perform 100ps MD operation, and observe the convergence of the density curve to obtain a stable simulated structure; finally, using the above-mentioned simulated structure as the initial configuration, adopt the NVT ensemble with a step size of 1fs, set the temperature to 298 K, and choose Andersen temperature control method [3], Atom Based and Ewald methods [4] are used to calculate the van der Waals force and electrostatic force respectively, and

300ps dynamical simulations are carried out, and finally the last 100 results of the trajectory file are extracted for analysis.

1.1. Binding Energy Analysis

The binding energy (E_{bind}) between the significant crystalline surfaces of NTO and HMX with the four polymer coating molecules was calculated according to the following equation:

$$E_{bind} = -E_{inter} = -[E_{total} - (E_{crystal} + E_{polymer})] \quad (S1)$$

E_{bind} is the binding energy between the polymer coating molecules and the important crystalline surfaces; E_{inter} is the interaction energy between the polymer and the important crystalline surfaces; E_{total} is the energy of the coating system derived from the equilibrium structure; $E_{crystal}$ is the energy of the coating system with the polymer removed from the explosive; $E_{polymer}$ is the energy of the coating system with the polymer removed from the main explosive. Finally, the final results are obtained by extracting the average energy of the last 100 structures in the trajectory file with a Perl script.

1.2. Cohesive Energy Density (CED) Analysis

The cohesive energy density (CED) is the sum of van der Waals (vdW) and electrostatic force, which is essentially a non-bonding force. CED reflects the strength of intermolecular interactions in a system. Generally speaking, the higher the polarity of the group, the stronger the intermolecular force and the higher the CED value.

CED is calculated as:

$$CED = (H_v - RT) / V_m \quad (S2)$$

where H_v is the molar heat of evaporation, RT is the expansion work done during the vaporization process, and V_m is the molar volume. Based on the trajectory file of the MD simulation, the CED of each system can be measured.

1.3. Mechanical Properties Analysis

The material generates internal stresses and strains in response to external stimuli, both satisfying Hooke's law, and the elasticity coefficient C_{ij} reflects the elastic effect of the material at different locations[5]. The matrix of elastic coefficients is obtained from the first-order partial derivatives of the corresponding components of the generalized Hooke's law equation, whose number decreases as the symmetry of the system increases. For isotropic materials, they have only two independent elasticity coefficients C_{11} and C_{12}. Therefore, define $C_{12} = \lambda$ and C_{11}-$C_{12} = 2\mu$ and call them lame coefficients. The tensile stress-strain curve is fitted by least squares to obtain the average value of the tensile modulus (or Young's modulus) and to obtain other valid isotropic mechanical properties such as tensile modulus (E), shear modulus (G) and bulk modulus (K).

References

1. Sun, H.J. COMPASS: An ab initio force field optimized for condensed-phase applications overview with details on alkane and benzene compounds. *J. Phys. Chem. B* **1998**, *102*, 7338.
2. Berendsen, H. J. C.; Postma, J. P. M.; van Gunsteren, W. F.; DiNola, A.; Haak, J. R. Molecular-dynamics with coupling to an external bath. *J. Chem. Phys*. **1984**, *81*, 3684.
3. Andersen, H.C. Molecular dynamics simulations at constant pressure and /or temperature. *J. Chem. Phys*. **1980**, *72*, 2384.
4. Karasawa, N.; Goddard, W. Force fields, structures, and properties of poly(vinylidene fluoride) crystals. *Macromolecules* **1992**, *25*, 7268.
5. Hang, G.Y.; Yu, W.L.; Wang, T.; Wang, J.; Li, Z. Theoretical investigations on stabilities, sensitivity, energetic performance and mechanical properties of CL-20 /NTO cocrystal explosives by molecular dynamics simulation. *Theor. Chem. Acc*. **2018**, *137*, 14.
